# Sparking nurses’ creativity: the roles of ambidextrous leadership and psychological safety

**DOI:** 10.1186/s12912-024-02277-1

**Published:** 2024-09-11

**Authors:** Heba Emad El-Gazar, Nadiah A. Baghdadi, Sally Mohammed Farghaly Abdelaliem, Mohamed Ali Zoromba

**Affiliations:** 1 Nursing Administration Department, Faculty of Nursing, Port Said University, Port Said, Egypt; 2 Nursing Management and Education Department, College of Nursing, Princess Nourah bint Abdulrahman University, P.O. BOX 84428, Riyadh, 11671 Saudi Arabia; 3 Nursing Department, College of Nursing, Prince Sattam bin Abdulaziz University, Al-Kharj, Saudi Arabia; 4 Psychiatric and Mental Health Nursing Department, Faculty of Nursing, Mansoura University, Mansoura, Egypt

**Keywords:** Ambidextrous Leadership, Creativity, Nurses, Psychological safety

## Abstract

**Background:**

The leadership practices of nurse managers significantly impact the creativity of staff nurses; however, the effects of ambidextrous leadership on nurses’ creativity are not yet clear. Additionally, the underlying mechanism of this relationship remains to be identified.

**Aim:**

The study aimed to examine the effect of ambidextrous leadership on nurses’ creativity, directly and indirectly through psychological safety.

**Methods:**

In this cross-sectional study, data were collected from October 2023 to January 2024 involving 241 nurses working at three hospitals in Port Said, Egypt. The Ambidextrous Leadership Scale, Psychological Safety Scale, and the Individual Creativity Scale were employed. Descriptive analysis, correlation analysis, and structural equation modeling were conducted.

**Results:**

Nurse managers’ ambidextrous leadership was positively associated with nurses’ creativity. Psychological safety fully mediated the association between ambidextrous leadership and nurses’ creativity.

**Conclusion:**

The study suggests that enhancing the ambidexterity of nurse leaders can foster a sense of psychological safety, which, in effect, contributes to increased creativity among nurses.

**Implication for nursing policymaking:**

There is a need for healthcare policies and strategies that are supportive of the implementation of ambidextrous leadership practices and promote psychological safety among nurses.

## Introduction

Healthcare organizations are currently experiencing a significant period of transformation driven by rapid technological advancements and shifting societal needs [1]. Consequently, it is crucial for healthcare leaders, particularly nurse managers, to maintain a balance between adopting innovative approaches and enhancing existing practices to deliver high-quality care [2]. Achieving this balance can be facilitated through the principle of ambidexterity [3], which entails leveraging established capabilities while simultaneously seeking new possibilities and integrating them with flexibility [4].

The academic nursing literature on ambidexterity has demonstrated that the ambidextrous behaviors of nurse managers are crucial for effectively managing current patient care while adapting to future changes [5]. Additionally, engaging nurse managers in ambidextrous leadership practices enhances their clinical leadership and increases work engagement among nursing staff [6]. However, to the best of the authors’ knowledge, it remains uncertain whether ambidextrous leadership fosters creativity among nurses. Given the pivotal role of nurses’ creativity in advancing the nursing sector, enhancing care quality, and improving patient outcomes [7], it is essential to elucidate the determinants of nurses’ innovative behaviors [8]. Therefore, investigating whether the ambidexterity behaviors of nurse managers can directly spark creativity in nurses is imperative, as this relationship has not yet been explored within the context of nursing.

Furthermore, this study aims to enhance understanding of the mechanisms that associate nurse managers’ ambidextrous leadership with nurses’ creativity. Although academic nursing inquiries have captured numerous consequential aspects of nurse managers’ ambidextrous leadership, we contend that these studies have neglected to explore how ambidextrous leadership produces its effects on nurses. Hence, the present study seeks to examine the effect of ambidextrous leadership on nurses’ creativity, directly and indirectly through psychological safety.

## Literature review and hypotheses development

### Ambidextrous leadership

Ambidextrous leadership refers to the ability of leaders to simultaneously invest in current services and explore new opportunities for the future [9]. In the field of nursing, Hannah et al. characterize the ambidexterity of nurse managers as the dual capacity to manage ongoing patient care processes (production-oriented) while pursuing innovative care processes for future needs (development-oriented) [10]. This concept requires flexible trade-offs between two leadership strategies: exploration and exploitation. Exploration involves seeking new opportunities and experimenting with innovative ideas, whereas exploitation focuses on enhancing and perfecting existing processes and services to optimize efficiency [3]. Previous literature indicates that nurse managers’ ambidextrous leadership is positively associated with nurses’ work-to-family enrichment and improved mental health [11]. Nurses who report to an ambidextrous leader also tend to perform better in their roles, provide higher quality services [12], and demonstrate greater engagement in their work [6].

### Ambidextrous leadership and creativity

Creativity is defined as the capacity to initiate novel or unprecedented elements within the nursing profession [13]. In healthcare, creativity refers to the process of implementing new and improved ideas to achieve better health promotion, disease prevention, and patient care [14]. Similarly, in nursing, creativity is defined as the development of new nursing practices to replace traditional ones or the improvement of existing practices [15]. To foster creativity within an organization, it is crucial for leaders to create an environment that encourages employees to take risks and engage in experimental trials [16]. Ambidextrous leadership is characterized by an openness to new ideas, a willingness to take risks, and a readiness to experiment with new opportunities [4]. Additionally, the literature on creativity suggests that creativity often emerges when individuals are confronted with contradictions and paradoxes [17]. Leaders who practice ambidextrous leadership effectively navigate between two seemingly contradictory approaches—exploration and exploitation [18]. Therefore, in applying this framework to nursing, we hypothesize:

H1. The ambidextrous leadership of nurse managers has a direct positive effect on nurses’ creativity.

### Mediating role of psychological safety

Psychological safety is the confidence to act authentically, unafraid of damaging one’s self-image or hindering career advancement [19]. Research indicates significant benefits of psychological safety among nurses; for instance, those with higher levels of psychological safety demonstrate increased job satisfaction, decreased intentions to leave their positions, and improved patient safety [20]. This study argues that ambidextrous leadership—characterized by the ability to effectively balance between the distinct leadership styles of exploration, which involves openness to search, risk-taking, and experimentation, and exploitation, which focuses on refining and perfecting existing processes to optimize efficiency [3]—can significantly enhance psychological safety within staff. The ability of leaders to adeptly switch between these styles, according to situational demands they encounter [21], builds trust in their leadership capabilities and confidence in their mastery. This, in turn, bolsters the psychological safety perceived by employees [22], thereby enabling them to embrace risks and cultivate an open, explorative mindset [23] that can ultimately lead to heightened creativity [24]. Therefore, in applying this framework to nursing, we hypothesize:

H2. The ambidextrous leadership of nurse managers has a direct positive effect on nurses’ psychological safety.

H3. The ambidextrous leadership of nurse managers has an indirect effect on nurses’ creativity via the mediating role of psychological safety.

**Fig. 1:**
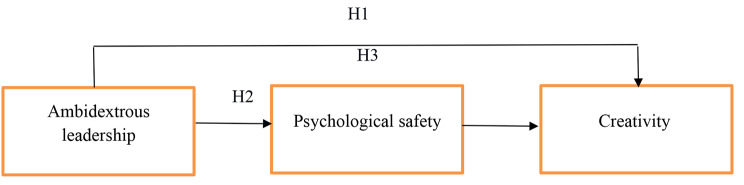
Conceptual model

Conceptual model depicted in Fig. 1.

## Subjects and methods

### Study design

We conducted a descriptive, cross-sectional study in accordance with the STROBE guidelines, seeking to examine the effect of ambidextrous leadership on nurses’ creativity, directly and indirectly through psychological safety.

### Participants and setting

We recruited inpatient ward nurses from three hospitals in Port Said, Egypt, two of which were health insurance hospitals, and one was a public hospital. Participants were selected through a three-stage sampling method. Firstly, random sampling was employed to pick those three hospitals from the list of hospitals within the region. Secondly, we employed stratified sampling to determine the number of nurses needed from each hospital. Thirdly, the nurses were selected conveniently from each hospital. The inclusion criteria were: (a) possession of a registered nurse license; (b) employment for at least 12 months and working under the current direct nurse manager for at least 6 months; (c) willingness to participate. Trainee nurses or nurses in managerial positions were excluded.

The sample size for our study was determined using a prior sample size calculation for structural equation modeling (SEM) software [25]. This calculation was based on a power level of 0.95, an anticipated effect size of 0.3, a desired probability level of 0.01, the presence of three latent variables, and 34 observed variables, which yielded a minimum required sample of 237 participants. To account for a potential 20% failure rate, we initially recruited 285 participants. Out of these, we received 253 responses and identified 12 as invalid, resulting in a final sample size of 241 nurses; a valid response rate of 84.6%.

### Instruments

Scales employed in the current study were translated from English to Arabic via a translation-back-translation procedure [26]. Specifically, two bilingual research assistants first translated the original English items into Arabic. Then, a third research assistant translated the items back into English. We collaborated to resolve any discrepancies between the original and the back-translated versions. Following this, a panel of seven experts, including four nursing professors, one nursing director, and two ward nurses with master’s degrees in nursing, reviewed the translated version alongside the original to ensure equivalence of terms and verify that the language was clear and straightforward. Some words were modified to better suit the context of Egyptian nurses. Before the final administration, a pre-study involving 24 nurses was conducted to ensure the comprehensibility of the questionnaire. Unless otherwise specified, a five-point Likert scale was used to assess each measure, ranging from “1 = strongly disagree” to “5 = strongly agree.”

#### Demographic characteristics

Demographic information for each participant, including age, gender, marital status, education, and years of working in nursing and in the current unit, was collected.

#### Ambidextrous leadership

The 14-item Managers’ Ambidexterity Scale [3] was utilized to examine nurses’ perceptions of their nurse managers’ ambidextrous leadership behaviors. This scale comprises two subscales: exploration leadership behaviors and exploitation leadership behaviors, each containing seven items. A sample item is “My managers focus on strong renewal of services or processes.” Participants responded using a 7-point scale, ranging from “1 = to a very small extent” to “7 = to a very large extent,” where higher scores indicate that nurses perceive a higher level of their nurse managers’ ambidextrous leadership practices. The findings of the confirmatory factor analysis (CFA) in this study demonstrated a satisfactory fit: χ2/df = 2.47, RMSEA = 0.078, TLI = 0.95, IFI = 0.96, CFI = 0.96.

#### Psychological safety

The seven-item Psychological Safety Scale [19] was used to assess nurses’ level of psychological safety. A sample item is “It is safe to take a risk on this team.” Higher scores indicate greater psychological safety. The findings of the CFA in this study demonstrated a satisfactory fit: χ2/df = 2.31, RMSEA = 0.074, TLI = 0.98, IFI = 0.98, CFI = 0.98.

#### Creativity

The 13-item Individual Creativity Scale [27] was employed to measure nurses’ level of creativity. A sample item is “Suggests new ways to increase quality.” Higher scores indicate greater creativity. The findings of the CFA in this study demonstrated a satisfactory fit: χ²/df = 2.45, RMSEA = 0.078, TLI = 0.94, IFI = 0.95, CFI = 0.95.

### Pre-study

We conveniently recruited 24 nurses from study hospitals to conduct a pre-study; these nurses were not included in the subsequent full study. The objective of the pre-study was to evaluate the understandability and clarity of the study questionnaires. The respondents confirmed that the questionnaires were clear and understandable, and no modifications were deemed necessary. In this pre-study, the Cronbach’s alpha values were 0.93 for the Ambidextrous Leadership Scale, 0.94 for the Psychological Safety Scale, and 0.91 for the Individual Creativity Scale.

### Procedure

Data was gathered from October 2023 to January 2024, following approval from hospital management and after securing the cooperation of head nurses in each ward. Copies of the questionnaire, along with envelopes, were distributed to nurses on-site during the morning shift. Each questionnaire was accompanied by a cover page that outlined the purpose and nature of the study and emphasized the principles of anonymity, confidentiality, and voluntary participation. To maintain anonymity, respondents were instructed to place their completed questionnaires in the provided envelopes, seal them, and individually return them to the researchers within three days.

### Common method variance (CMV)

Considering the use of a cross-sectional research design in this study, several procedural remedies were implemented to mitigate common method variance (CMV). Specifically, scale items were subjected to expert review and pre-testing. Participants were informed that the research was conducted for academic purposes and that their names were not required. Additionally, the questionnaire included items with varying scale formats, and these items were randomly distributed throughout the questionnaire [28]. It was also essential to assess the extent to which CMV might influence the findings. Following the recommendations of Podsakoff et al., we employed Harman’s one-factor test to evaluate the presence of CMV [29]. The analysis indicated that the first factor explains 42.54% of the variance, which is below the threshold of 50% [30].

### Data analysis

Analyses in this study were conducted using SPSS 28.0 and Amos 25.0 software. Descriptive statistics were utilized to summarize the demographic characteristics and scores of the study variables. The differences in nurses’ demographic characteristics related to primary study variables were examined using independent t-tests and analysis of variance (ANOVA). Pearson correlation analysis was employed to investigate the relationships between the study variables. The reliability and validity of the study measures were confirmed. SEM was used to test the measurement model and to explore the mediating role of psychological safety in the relationship between ambidextrous leadership and nurses’ creativity. The goodness-of-fit indices were assessed according to the criteria of χ2/df < 3, RMSEA < 0.08, and TLI, IFI, and CFI ≥ 0.90 [31]. Statistical significance was determined at a two-tailed p-value of < 0.05.

## Results

### Participants’ characteristics

**Table 1:** Participants’ characteristics and comparison of the study variables (*N* = 241)

Variable	*n* (%)	Ambidextrous leadership		Psychological safety		Creativity	
		Mean (SD)	t/F (P)	Mean (SD)	t/F (P)	Mean (SD)	t/F (P)
**Age (years)**; mean ± SD (31.72 ± 7.42)							
≤ 30	147 (61.0)	4.21 (0.92)	t = -1.23 (0.222)	3.71 (0.89)	t = -0.18 (0.858)	3.46 (1.06)	t = -1.67 (0.097)
> 30	94 (39.0)	4.35 (0.83)		3.73 (0.88)		3.60 (0.88)	
**Gender**							
Male	68 (28.2)	4.12 (0.99)	t = -1.54 (0.125)	3.72 (0.88)	t = 0.53 (0.958)	3.61 (0.87)	t = 0.89 (0.372)
Female	173 (71.8)	4.32 (0.84)		3.71 (0.89)		3.50 (0.90)	
**Marital status**							
Single	33 (13.7)	3.95 (0.97)	F = 1.57 (0.197)	3.71 (0.84)	F = 0.34 (0.797)	3.49 (0.90)	F = 0.06 (0.981)
Married	188 (78.0)	4.32 (0.88)		3.69 (0.89)		3.53 (0.89)	
Divorced	9 (3.7)	4.26 (0.82)		4.00 (1.09)		3.59 (1.19)	
Widowed	11 (4.6)	4.31 (0.53)		3.75 (0.76)		3.61 (0.86)	
**Education**							
Diploma	89 (36.9)	4.20 (0.85)	F = 1.97 (0.141)	3.69 (0.91)	F = 0.15 (0.861)	3.61 (0.96)	F = 1.98 (0.140)
Associate	95 (39.4)	4.20 (0.91)		3.70 (0.86)		3.39 (0.85)	
Bachelor/higher	57 (23.7)	4.47 (0.89)		3.77 (0.90)		3.65 (0.87)	
**Years in nursing;** mean ± SD (11.52 ± 7.28)							
≤ 10	133 (55.2)	3.31 (0.79)	t = -0.95 (0.345)	3.22 (0.96)	t = 0.22 (0.826)	3.30 (1.04)	t = -1.11 (0.267)
> 10	108 (44.8)	3.38 (0.85)		3.43 (0.88)		3.26 (0.92)	
**Years in the current unit**; mean ± SD (5.63 ± 3.23)							
≤ 5	139 (57.7)	4.26 (0.92)	t = -0.11 (0.909)	3.70 (0.85)	t = -0.29 (0.765)	3.49 (0.87)	t = -0.69 (0.488)
> 5	102 (42.3)	4.27 (0.85)		3.73 (0.93)		3.58 (0.93)	

Note: t = independent sample t test, F = test of variance (ANOVA).

The majority of the study participants were female (71.8%), married (78.0%), and held an associate degree in nursing (39.4%), with a mean age of 31.72 years (SD = 7.42). The mean experience of the participants in nursing was 11.52 years (SD = 7.28), while their experience in their current unit was 5.63 years (SD = 3.23). There were no significant differences in participants’ demographics across the study variables (Table 1).

### Measurement model

**Table 2:** Descriptive statistics, reliability, validity, and correlations among study variables (*N* = 241)

Variable	Mean ± SD	α	Factor loadings	CR	AVE	1	2	3
1. Ambidextrous leadership	4.26 ± 0.89	0.95	0.77–0.89	0.87	0.77	**0.88**	*0.58*	*0.50*
2. Psychological safety	3.71 ± 0.88	0.93	0.77–0.85	0.93	0.65	0.55***	**0.81**	*0.68*
3. Creativity	3.53 ± 0.90	0.94	0.64–0.81	0.94	0.56	0.48***	0.64***	**0.75**

AVE, average variance extracted; CR, composite reliability; SD, standard deviation.

Bolded values on the diagonals represent the square root of AVE; off-diagonal values are construct correlations; Italics values represent HTMT.

***P < 0.001

To verify the validity of the measurement model, a series of CFAs were conducted. The results showed that the three-factor model, encompassing ambidextrous leadership, psychological safety, and creativity, demonstrated a satisfactory fit (χ²/df = 1.68, RMSEA = 0.053, TLI = 0.94, IFI = 0.95, CFI = 0.94), in comparison to the two-factor model that combined ambidextrous leadership and psychological safety (χ²/df = 3.96, RMSEA = 0.111, TLI = 0.74, IFI = 0.76, CFI = 0.76), and the one-factor model that merged all variables (χ²/df = 6.26, RMSEA = 0.148, TLI = 0.54, IFI = 0.57, CFI = 0.56).

The convergent and discriminant validity of the study measures were assessed. Convergent validity was evaluated through a check of factor loadings, construct reliability (CR), and average variance extracted (AVE) values. For all study measures, all indicator factor loadings (ranging from 0.64 to 0.89) were higher than the recommended value of 0.5; the CR values (ranging from 0.87 to 0.94) exceeded the benchmark of 0.7; and the AVE (ranging from 0.56 to 0.77) was above the acceptable level of 0.5 [32], providing evidence for convergent validity. The discriminant validity was evaluated by a check of HTMT estimates, and comparing the square roots of the AVE with the variance shared between the constructs. HTMT values (ranging from 0.50 to 0.68) were below the cut-off point of 0.85 [33]; and the square roots of the AVE for the three constructs exceeded the inter-construct correlations, providing good discriminant validity [34]. Additionally, Cronbach’s alphas for all instruments (ranging from 0.93 to 0.95), exceeded the recommended 0.70 [35], demonstrating acceptable internal consistency for the study instruments (Table 2).

### Preliminary analysis

Means, standard deviations, and correlations among variables are presented in Table 2. The mean scale scores for ambidextrous leadership, psychological safety, and creativity were 4.26 (SD: 0.89), 3.71 (SD: 0.88), and 3.53 (SD: 0.90), respectively. These mean scores suggest that the participating nurses perceived their direct nurse managers to have a moderate level of ambidexterity, while reporting relatively high levels of psychological safety and creativity. Additionally, there was a significant positive relationship between ambidextrous leadership and nurses’ psychological safety (*r* = 0.55, *p* < 0.01), and creativity (*r* = 0.48, *p* < 0.01). Also, there was a significant positive relationship between nurses’ psychological safety and creativity (*r* = 0.64, *p* < 0.01).

### Hypotheses testing

**Table 3:** Results of the mediation model (*N* = 241)

Path	β	SE	t	*P*	95% confidence interval
					Lower/Upper
AL to psychological safety	0.63	0.07	7.65	< 0.001	0.53/0.72
Psychological safety to creativity	0.61	0.08	7.14	< 0.001	0.45/0.74
AL to creativity	0.12	0.06	1.57	0.116	-0.04/0.30
Indirect effect of AL on creativity	0.38				0.29/0.49
Total effect	0.50				0.36/0.62

AL, ambidextrous leadership.

**Fig. 2:**
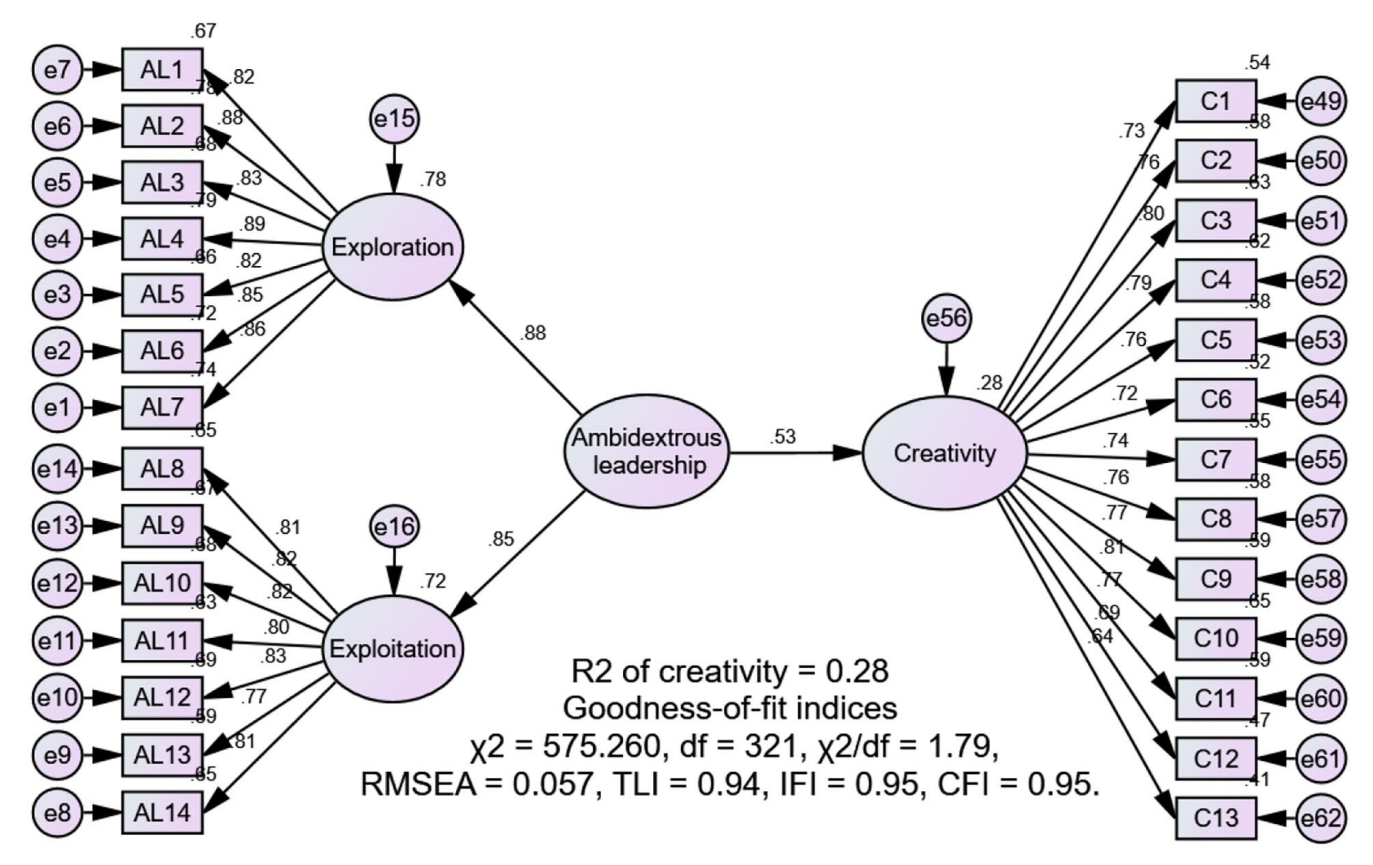
Direct link model between ambidextrous leadership and creativity

**Fig. 3:**
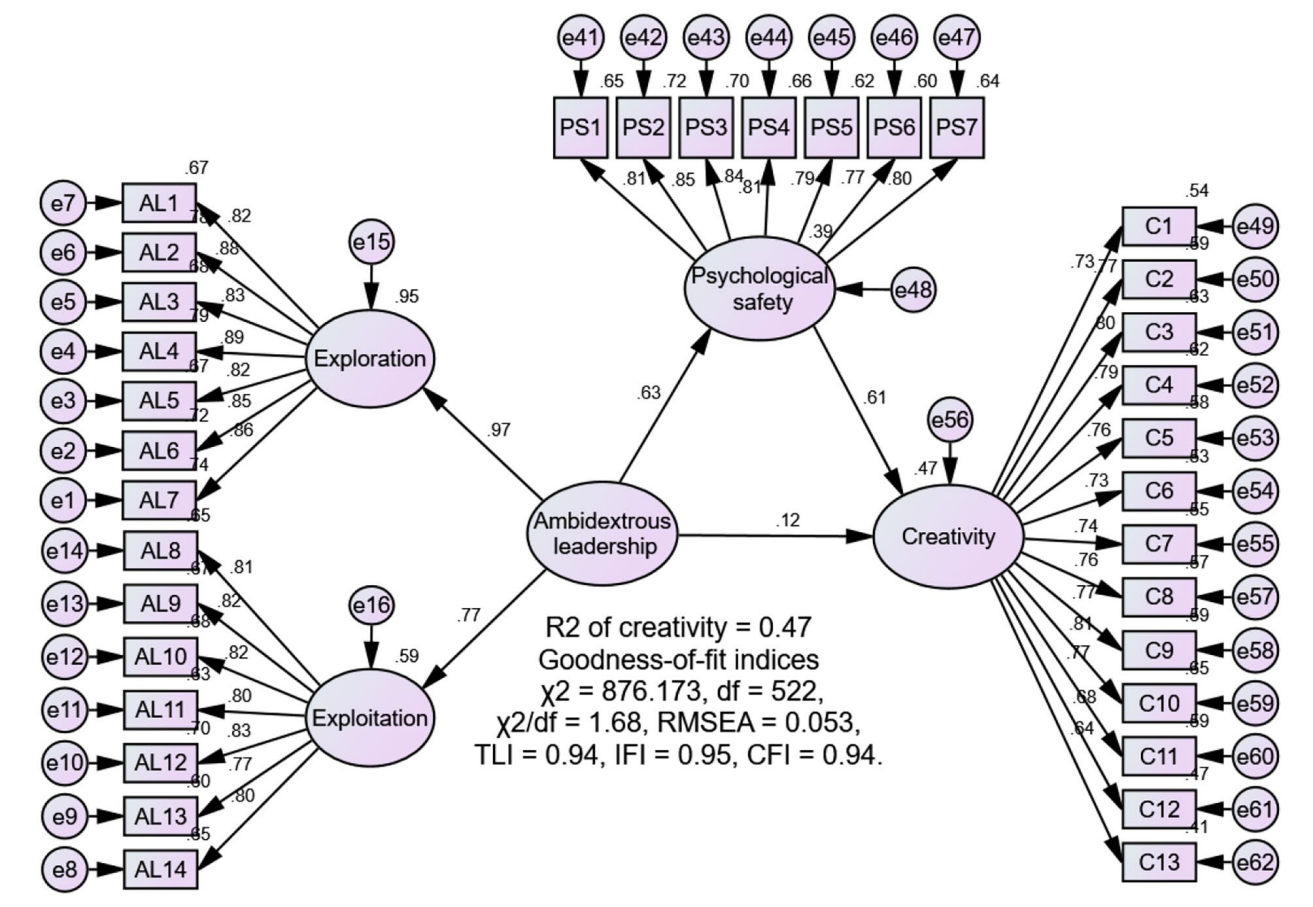
Mediation model

We applied SEM to validate the study’s hypotheses. First, the direct effect of ambidextrous leadership on nurses’ creativity was examined. The results indicated that ambidextrous leadership significantly contributed to nurses’ creativity (β = 0.53, *p* < 0.001), accounting for 28% of the variance in nurses’ creativity with a good fit of the model: χ²/df = 1.79, RMSEA = 0.057, TLI = 0.94, IFI = 0.95, CFI = 0.95, thus supporting H1 (Fig. 2). Subsequently, we assessed the mediating effect of nurses’ psychological safety in the relationship between ambidextrous leadership and creativity using 5000 bootstrap samples and 95% confidence intervals. The mediation model exhibited a satisfactory fit: χ²/df = 1.68, RMSEA = 0.053, TLI = 0.94, IFI = 0.95, CFI = 0.94. Ambidextrous leadership was found to positively contribute to nurses’ psychological safety (β = 0.63, *p* < 0.001), confirming H2. In addition, psychological safety positively contributed to nurses’ creativity (β = 0.61, *p* < 0.001). The indirect effect of ambidextrous leadership on creativity was significant (β = 0.38, 95% confidence interval: 0.29–0.49). Furthermore, the relationship between ambidextrous leadership and creativity became non-significant when psychological safety was added in the model (β = 0.12, *p* = 0.116). These findings support the full mediation of psychological safety in the relationship, thus confirming H3. Together, ambidextrous leadership and psychological safety explained 47.0% of the variance in nurses’ creativity (Fig. 3; Table 3).

## Discussion

This study investigated the effect of ambidextrous leadership on nurses’ creativity, directly and indirectly through psychological safety. Initially, the findings demonstrated that nurse managers’ ambidextrous leadership can significantly enhance nurses’ creativity. Ambidextrous leaders, capable of employing open leadership behaviors [36], encourage nurses to perform their work in unique ways, incorporating diverse ideas, which assists them in meeting the creative demands of the innovation process [37]. In the nursing field, these results align with prior studies that have shown how ambidexterity can foster innovative behaviors [38]. These findings are also consistent with a non-nursing study by Cheng, which suggests that ambidextrous leadership contributes to the innovative behaviors of followers [39]. Additionally, these results are congruent with findings indicating that ambidextrous leadership encourages employees to express new ideas [40].

Furthermore, the study findings revealed that nurse managers’ ambidextrous leadership can effectively enhance nurses’ psychological safety. Ambidextrous leaders are characterized by their flexibility to shift between two behaviors: exploration and exploitation [41]. Exploration leadership behaviors boost idea generation by encouraging unconventional thinking, promoting experimentation, fostering risk-taking, and supporting learning from errors [18]. Conversely, exploitation leadership behaviors facilitate idea implementation within the constraints posed by organizational features [3]. The interplay between these two behaviors fosters a sense of equilibrium, which in turn promotes a feeling of safety among nurses [42]. These results are similar to those reported in the non-nursing context, where ambidexterity on the part of leadership has been found to promote followers’ psychological safety [43].

In addition, the study findings showed that the psychological safety of nurses can effectively increase their creativity. This may be attributed to nurses with a sense of psychological safety feeling comfortable expressing their ideas, sharing opinions, and engaging in risk-taking behaviors [19]. Such an environment could motivate nurses to engage in creative thinking, which ultimately enhances their creativity. These results align with a recent study conducted among healthcare professionals, which demonstrated that when health professionals experience psychological safety, they become more creative in their work [44]. Additionally, Zhao et al. showed that ambidextrous leadership practices can enhance followers’ psychological safety, further supporting the link between ambidextrous leadership and creativity [45].

Lastly, the findings of this study suggest that psychological safety serves as a mediator in the relationship between the ambidextrous leadership of nurse managers and the creativity of nurses. This implies that when nurses perceive their managers as receptive to process improvements for better patient outcomes while effectively managing daily operations, they feel secure in expressing their ideas and taking charge without fear of retaliation. This sense of security can, in turn, spark the creativity of nurses. These findings corroborate prior research in non-nursing contexts, which indicates that positive [44] and situational leadership [46] behaviors can foster followers’ creativity by enhancing psychological safety.

### Limitations

The study had several limitations worth mentioning. Firstly, data collection from one city in Egypt restricts the generalizability of the findings. Future research should aim to expand the geographical scope of data collection to enhance the external validity of the results. Secondly, employing a cross-sectional design restricts the capacity to determine causal relationships. Future investigations should employ longitudinal designs to more effectively ascertain causality. Lastly, reliance on self-reported data introduces the possibility of social desirability bias. This issue underscores the necessity for future studies to utilize alternative methods of data collection to reduce this bias.

### Implications

The study enriches nursing science in three notable ways. First, it builds upon previous research regarding the outcomes of nurse managers’ ambidextrous leadership. Second, it deepens theoretical understanding of the factors influencing nurses’ creativity. Third, it introduces psychological safety as a mediating mechanism in the relationship between nurse managers’ ambidextrous leadership and the enhancement of nurses’ creative behaviors.

This study offers several implications for healthcare organizations and nursing management. The findings underscore the importance of cultivating ambidextrous leadership among nursing managers to enhance nurses’ creativity. As a result, hospital administrators should focus on developing the ambidextrous leadership skills of nurse managers, particularly head nurses. Since ambidexterity can be taught [40], training programs can be designed to enhance nurse managers’ ability to balance explorative and exploitative behaviors, thus creating a supportive environment that fosters nurses’ creativity. Additionally, during the hiring process of nurse managers, hospital administrators should prioritize candidates who demonstrate resilience, encourage innovative ideas, and support the adoption of innovative practices within safe boundaries.

Furthermore, the study highlights the mediating role of psychological safety in the relationship between nurse managers’ ambidextrous leadership and the enhancement of nurses’ creative behaviors. Therefore, healthcare organizations should establish a culture in which nurses feel comfortable expressing their thoughts and taking risks without fear of negative consequences. This can be achieved through transparent communication, regular feedback, inclusive decision-making processes, and by fostering a culture that views mistakes as opportunities for learning.

## Conclusion

The study contributed novel insights to the field of nursing science by exploring the effect of ambidextrous leadership on nurses’ creativity, directly and indirectly through psychological safety. The findings indicated that nurse managers’ ambidextrous leadership can cultivate a psychological safety and creativity among nurses. Furthermore, the study supports the notion that psychological safety serves as a mediating mechanism through which the ambidextrous leadership of nurse managers can enhance nurses’ creativity.

## Data Availability

The datasets generated during and analyzed during the current study are not publicly available due to confidentiality agreements, but are available upon reasonable request from the corresponding author.
